# Leveraging a disulfidptosis-based signature to improve the survival and drug sensitivity of bladder cancer patients

**DOI:** 10.3389/fimmu.2023.1198878

**Published:** 2023-05-30

**Authors:** Hualin Chen, Wenjie Yang, Yingjie Li, Lin Ma, Zhigang Ji

**Affiliations:** Department of Urology, Peking Union Medical College Hospital, Chinese Academy of Medical Sciences and Peking Union Medical College, Beijing, China

**Keywords:** disulfidptosis, bladder cancer, molecular clusters, tumor immune microenvironment, prognostic model, POU5F1, CTSE

## Abstract

**Background:**

Disulfidptosis is a recently discovered form of cell death. However, its biological mechanisms in bladder cancer (BCa) are yet to be understood.

**Methods:**

Disulfidptosis-related clusters were identified by consensus clustering. A disulfidptosis-related gene (DRG) prognostic model was established and verified in various datasets. A series of experiments including qRT-PCR, immunoblotting, IHC, CCK-8, EdU, wound-healing, transwell, dual-luciferase reporter, and ChIP assays were used to study the biological functions.

**Results:**

We identified two DRG clusters, which exhibited distinct clinicopathological features, prognosis, and tumor immune microenvironment (TIME) landscapes. A DRG prognostic model with ten features (DCBLD2, JAM3, CSPG4, SCEL, GOLGA8A, CNTN1, APLP1, PTPRR, POU5F1, CTSE) was established and verified in several external datasets in terms of prognosis and immunotherapy response prediction. BCa patients with high DRG scores may be characterized by declined survival, inflamed TIME, and elevated tumor mutation burden. Besides, the correlation between DRG score and immune checkpoint genes and chemoradiotherapy-related genes indicated the implication of the model in personalized therapy. Furthermore, random survival forest analysis was performed to select the top important features within the model: POU5F1 and CTSE. qRT-PCR, immunoblotting, and immunohistochemistry assays showed the enhanced expression of CTSE in BCa tumor tissues. A series of phenotypic assays revealed the oncogenetic roles of CTSE in BCa cells. Mechanically, POU5F1 can transactivate CTSE, promoting BCa cell proliferation and metastasis.

**Conclusions:**

Our study highlighted the disulfidptosis in the regulation of tumor progression, sensitivity to therapy, and survival of BCa patients. POU5F1 and CTSE may be potential therapeutic targets for the clinical treatment of BCa.

## 1 Introduction

Bladder cancer (BCa) is the most common urological neoplasm, with a significant impact on public health worldwide. There were over 570,000 new cases and 210,000 deaths from BCa globally in 2020 (1, 2). BCa can be categorized into non-muscle-invasive and muscle-invasive BCa. While muscle-invasive BCa accounts for only about 30% of newly diagnosed cases, its aggressive nature, propensity for metastasis, drug resistance, and high rate of recurrence contribute to reduced cancer-specific survival after R0 resection (3, 4). Hence, elucidating the molecular mechanisms underlying the progression of BCa is of paramount importance.

Recently, Liu and colleagues uncovered a novel form of cell death, disulfidptosis, which has not been characterized previously (5). Disulfidptosis is induced by the accumulation of intracellular disulfides in glucose-starved cells with overexpressed SLC7A11 (6). Unlike ferroptosis and apoptosis, disulfidptosis is mediated by the susceptibility of the actin cytoskeleton to disulfide stress. The study also shows that glucose transporter inhibitors trigger disulfidptosis and control tumor proliferation, suggesting the significance of disulfidptosis in cancer management. Overall, the study sheds light on the role of disulfidptosis in controlling tumor progression.

In the study, we aimed to comprehensively investigate the role of DRGs in the prognosis, TIME landscapes, and drug resistance in BCa. We developed and validated a DRG-related prognostic model, which demonstrated high accuracy in predicting prognosis and response to immunotherapy across various independent cohorts. POU5F1 and CTSE were the top two important features within the model. Mechanistically, POU5F1 can transactivate CTSE and promote the proliferation and metastasis of BCa. These results suggested potential therapeutic targets for BCa treatment.

## 2 Methods

### 2.1 Data collection, tumor somatic mutation analysis, and protein-protein interaction analysis

The BCa datasets were obtained from the TCGA-BLCA and GEO databases (GSE13507, GSE32548, and GSE32894), as previously described (7). Additional immune checkpoint blockade (ICB) -treated datasets, including the Mariathasan BCa cohort (8), Braun RCC cohort (9), and Liu SKCM cohort (10), were obtained from original publications. The details of these included datasets were provided in **Table S1**. The details of DRGs were provided in **Table S2**.

Gene Ontology Biological Processes (GO-BP), KEGG, and cancer hallmarks were obtained from the MSigDB (https://www.gsea-msigdb.org/gsea/msigdb/) (11). Immunohistochemistry (IHC) images of proteins were procured from the Human Protein Atlas (https://www.proteinatlas.org/) (12).

The mutational landscape of all TCGA-BLCA patients and the mutation of DRGs were analyzed using the “maftool” package (13). Online tool GeneMANIA was employed to construct the PPI network of 14 DRGs (http://genemania.org/) (14).

### 2.2 Unsupervised consensus clustering, differential analysis, enrichment analysis, and TIME landscape estimation

Unsupervised consensus clustering analysis of the TCGA-BLCA cohort was conducted based on the expression profiles of 14 DRGs using the “ConsensusClusterPlus” package (15).

Differentially expressed genes (DEGs) between DRG-based molecular clusters were identified using the “limma” package (16). DEGs with P value (adjusted)< 0.01 and |logFC [fold change] | > 1 were considered significant. The details of DEGs were provided in **Table S2**.

The enrichment analysis (GO, KEGG, and gene set enrichment analysis [GSEA]) was employed *via* the “clusterProfiler” package as described previously (7, 17). The activity of GO-BP and KEGG terms for each sample was quantified by the “GSVA” package (18).

TIME scores including stromal, immune, and ESTIMATE scores were determined by the “ESTIMATE” package (19). The infiltration levels of 22 immune cell subsets were estimated by the “CIBERSORT” package (20).

### 2.3 Establishment and verification of a disulfidptosis−related prognostic model

The model was developed and validated as described in our previous study (21). In brief, the TCGA-BLCA dataset was considered as the training cohort, while the three GEO BCa datasets were external validation cohorts. Univariate Cox regression analysis was first used to identify overall survival (OS)-related DEGs (OS-DEGs). The details of OS-DEGs were provided in **Table S2**. The least absolute shrinkage and selection operator (LASSO) Cox regression analysis was then employed to reduce the dimensionality of high latitude data. The disulfidptosis-related predictive model was finally constructed using multivariate Cox regression analysis. Each BCa sample of TCGA-BLCA was assigned a DRG score using the formula: DRG score = $\sum_{i=1}^{n}ki*Xi$ , where k_i represents the regression coefficient and X_i represents the relative expression level of gene i.

BCa samples were divided into high- and low-risk groups according to the median value of the DRG scores. Kaplan-Meier curves with the log-rank test were utilized to determine the OS and progression-free survival (PFS) between the two groups. The performance of the model was estimated by the time-dependent receiver operating characteristic (ROC) analysis.

R packages “survival”, “survminer”, and “timeROC” were used in these analyses (22, 23).

### 2.4 Tissue samples

The study protocol was approved by the Institutional Ethics Committee of Peking Union Medical College Hospital and we performed these experiments adhered to the principles of the Declaration of Helsinki. Tumor tissues of BCa and adjacent para-cancerous tissues were procured following radical surgery and histological confirmation. Written informed consent was obtained from all participants.

### 2.5 Cell culture and transfection

SV-HUC-1, T24, 5637, J82, and RT4 were purchased from the Cancer Institute of the Chinese Academy of Medical Sciences (Beijing, China). Cells were cultured in complete RPMI 1640 or DMEM supplemented with 10% fetal bovine serum (FBS) and 1% penicillin/streptomycin (Beyotime, Shanghai, China).

The siRNAs compounds targeting CTSE (siCTSE-1: 5’- CAACUACUUGGAUAUGGAAUA- 3’; siCTSE-2: 5’ – CAAUCUUUCUCCAUUCAGUAU – 3’) were designed by GenePharma (Suzhou, China). Transfection was performed using lipofectamine 3000 (Invitrogen) following the manufacturer’s instructions. Overexpression plasmid pcDNA3.1-POU5F1 and corresponding empty vector were purchased from Obio Technology Corp.

### 2.6 qRT-PCR, immunoblotting, and IHC

These assays were performed according to our previous studies (7, 17, 24). The primer sequences for qRT-PCR analysis were listed in **Table S3**. The information on all the antibodies used in the study was presented in **Table S4**.

### 2.7 Dual-luciferase reporter assay and chromatin immunoprecipitation

These procedures were performed as previously described (25). In brief, wild-type (CTSE-WT) and mutant-type (CTSE-Mut) sequences of the POU5F1 binding site in the promoter of CTSE were designed and cloned into the luciferase vectors (Beyotime, Shanghai, China). The overexpression vector or empty vector of POU5F1 was co-transfected into plated cells. After being cultured for 48 hours, dual luciferase activities were then evaluated by Dual-Luciferase Reporter Gene Assay Kit (Beyotime, Shanghai, China) following the manufacturer’s standard.

ChIP assay was employed using ChIP Assay Kit (Beyotime, Shanghai, China) following the instructions. After the cross-link, the anti-POU5F1 antibody was used to immunoprecipitate the corresponding genomic sequences. Then the sequences were analyzed by qPCR assay. The primer sequences for the CTSE promoter were provided in **Table S1**.

### 2.8 CCK-8, wound-healing, and transwell invasion assays

These phenotypic assays were performed as described in our previous studies (7, 17).

### 2.9 Statistical analyses

R 4.1.2 and GraphPad Prism 9.2.0 were employed for all statistical analyses. The correlation between the two variables was determined by the Spearman correlation coefficient. Student’s T-Test or Mann–Whitney U tests were used for continuous variables. Chi-Square Tests were utilized for categorized variables. A two-tailed p-value< 0.05 was considered statistically significant.

## 3 Results

### 3.1 Genetic and transcriptional characteristics of DRGs

**Figure 1:**
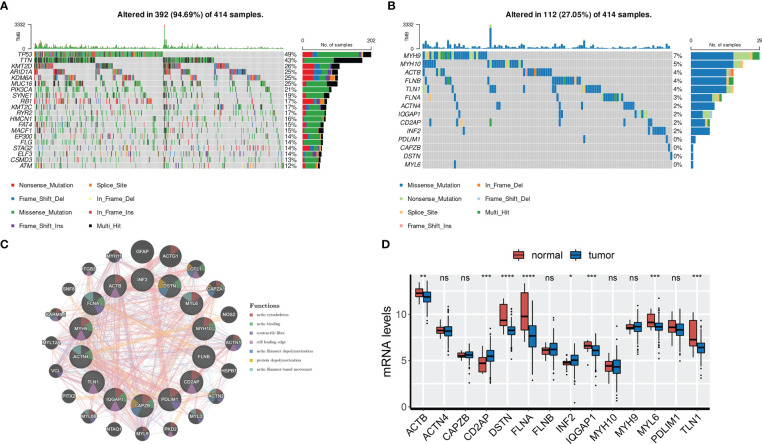
Genetic and transcriptional characteristics of DRGs. **(A)** Somatic mutation landscape of all patients. **(B)** Somatic mutation of 14 DRGs. **(C)** PPI network of 14 DRGs by GeneMANIA. **(D)** mRNA levels of 14 DRGs. ns, not significance, *p< 0.05, ***p< 0.001, ****p< 0.0001. ns, not significance.

First, we analyzed the somatic mutation landscapes of BCa patients, revealing that 94.69% of BCa samples showed somatic mutations (**Figure 1A**). Next, the somatic mutation pattern of 14 DRGs was also estimated (**Figure 1B**). Our results showed that DRGs in 112 out of 414 BCa samples (27.05%) had somatic mutations, primarily driven by missense mutations. In particular, MYH9, MYH10, ACTB, FLNB, and TLN1 showed the highest incidence of missense mutations, while MYH9 and ACTB had the highest incidence of nonsense mutations and in-frame deletions, respectively. To investigate the interactions between the 14 DRGs, the PPI network was constructed by the GeneMANIA online tool, which revealed that FLNA, MYH9, IQGAP1, CAPZB, and DSTN were regarded as the hub genes (**Figure 1C**).

We also analyzed the expression profiles of the 14 DRGs in the TCGA-BLCA cohort. Results showed that ACTB, DSTN, FLNA, IQGAP1, MYL6, and TLN1 were overexpressed in normal tissues, while CD2AP and INF2 were overexpressed in tumor tissues (**Figure 1D**). We further found that ACTB, DSTN, FLNA, and TLN1 were upregulated in high-grade and high-stage (stage III and IV) BCa tissues, indicating their association with BCa progression (**Figures S1A, B**). Finally, representative IHC images demonstrated the protein levels of the four genes (**Figure S1C**).

### 3.2 DRG-based molecular clusters with distinct clinical features and TIME landscapes

**Figure 2:**
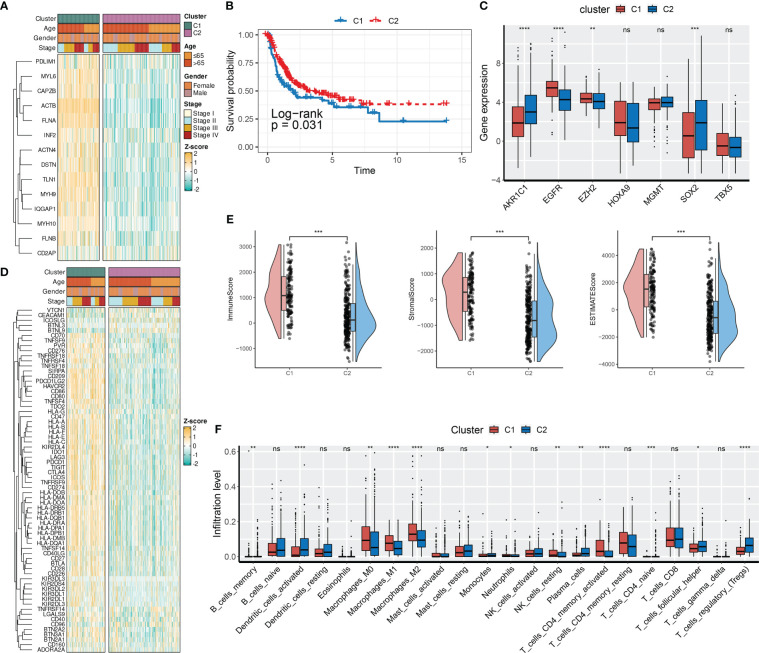
DRG-based molecular clusters with distinct clinical features and TIME landscapes. **(A)** Expression profiles of DRGs and clinicopathological characteristics between clusters. **(B)** Survival analysis between C1 and C2. **(C)** Expression levels of CRGs between clusters. **(D)** Expression profiles of ICGs and clinicopathological characteristics between clusters. **(E)** Differences in TIME scores between clusters. **(F)** Abundances of infiltrating immune cells between clusters. ns, not significance, *p< 0.05, **p< 0.01, ***p< 0.001, ****p< 0.0001. ns, not significance.

To investigate the biological roles of DRGs in BCa, we employed an unsupervised consensus clustering algorithm to categorize BCa samples of the TCGA-BLCA cohort based on the expression profiles of 14 DRGs. As indicated by the CDF curves and the PAC test, the optimal clustering number was 2 (**Figures S2A–C**). The expression levels of DRGs were higher in BCa samples of C1 compared to those of C2 (**Figure 2A**). In terms of clinical features, the proportion of patients with age > 65, male gender, and stage III and IV was relatively higher in C1 than in C2 (**Figures 2A**; **S2D–F**). Moreover, patients in C1 had a poorer prognosis compared to those in C2 (**Figure 2B**).

Next, we evaluated the expression levels of immune checkpoint genes (ICGs, **Table S5**) and chemoradiotherapy-related genes (CRGs, **Table S5**), as well as the TIME scores between two DRG-based clusters. As presented in **Figure 2C**, SOX2, EZH2, EGFR, and AKR1C1 of the CRGs had distinct expression patterns between the two DRG-based clusters. Specifically, AKR1C1 and SOX2 were downregulated in C1, whereas EGFR and EZH2 were overexpressed in C1. Regarding the TIME landscapes, we observed that samples of C1 had overexpressed ICGs (**Figure 2D**), a higher TIME score (**Figure 2E**), and higher infiltration levels of macrophages and activated memory CD4+ T cells (**Figure 2F**). However, samples of C2 had higher abundances of regulatory T cells, activated dendritic cells, monocytes, infiltrated memory B cells, and neutrophils.

### 3.3 DRGs-based molecular clusters with dysregulated pathways and biological process

**Figure 3:**
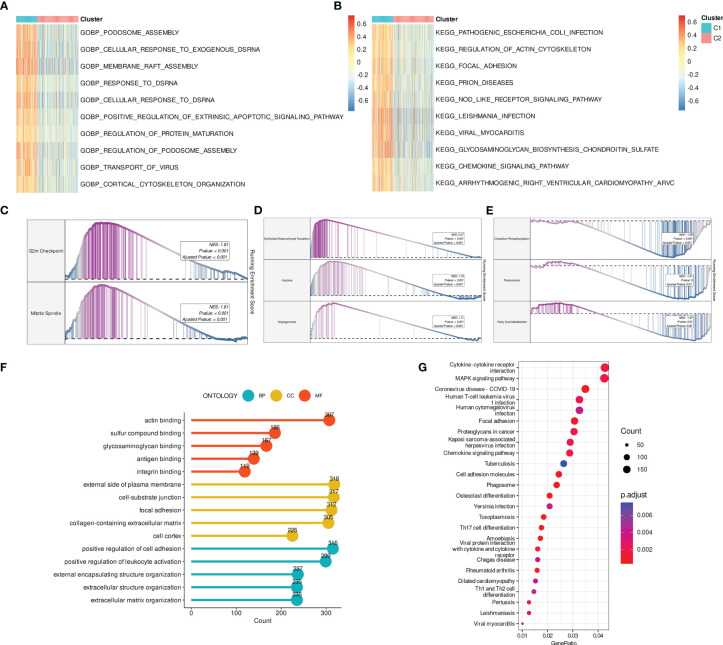
DRGs-based molecular clusters with dysregulated pathways and biological process. **(A, B)** GSVA of GO-BP **(A)** and KEGG **(B)** terms between clusters. **(C–E)** GSEA of significant hallmarks enriched in DRG C1 **(C, D)** and C2 **(E)**. **(F, G)** GO **(F)** and KEGG **(G)** analysis of cluster DEGs.

To investigate the pathways and biological processes between the two DRG clusters, a series of enrichment analyses were employed, including GSVA, GSEA, and over-representation analysis (ORA). The results of GO-BP GSVA showed that DRG C1 was enriched in cellular structure-related processes, including podosome assembly, membrane raft assembly, regulation of protein maturation, and cortical cytoskeleton organization (**Figure 3A**). KEGG term results revealed that DRG C1 was abundant in immunity-related pathways, including focal adhesion and NOD-like receptor signaling pathway (**Figure 3B**). GSEA subsequently uncovered that DRG C1 was significantly linked to cancer hallmarks, including the cell cycle (G2M checkpoint and mitotic spindle, **Figure 3C**) and cancergenesis and progression (epithelial-mesenchymal transition [EMT], hypoxia, and angiogenesis, **Figure 3D**). In contrast, DRG C2 was associated with metabolism, specifically oxidative phosphorylation and fatty acid metabolism (**Figure 3E**).

We then identified DRGs cluster-related DEGs to verify the findings above. Consistently, cellular structure and immunity-related biological processes and pathways were mainly enriched (**Figures 3F, G**).

### 3.4 Detection of gene clusters related to disulfidptosis in BCa

**Figure 4:**
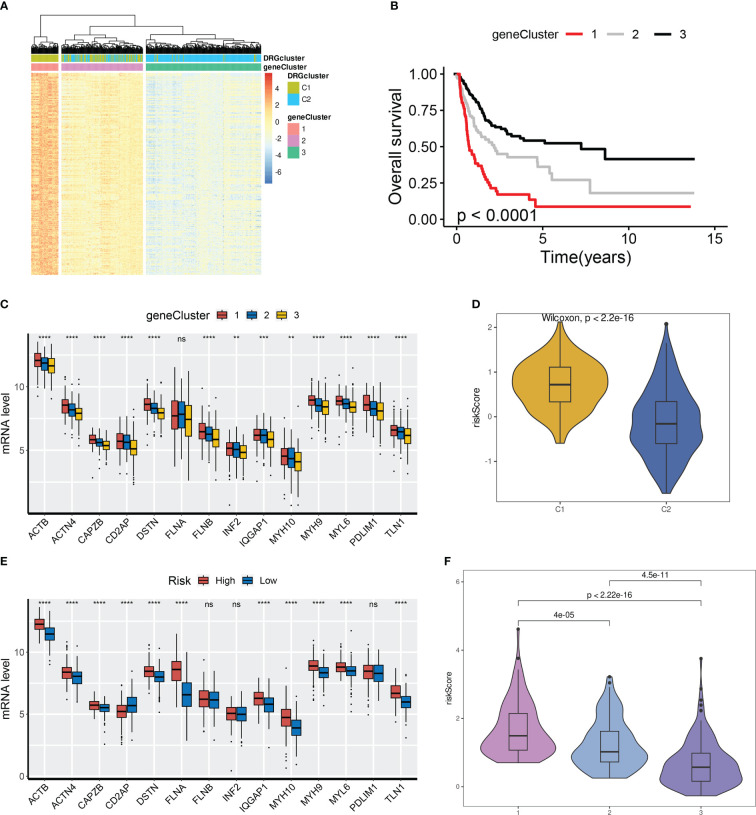
Detection of gene clusters related to disulfidptosis. **(A)** The expression profiles of DEGs and the distribution of DRG clusters among gene clusters 1 to 3. **(B)** Survival analysis among three gene cluster. **(C)** mRNA levels of 14 DRGs among gene clusters 1 to 3. **(D)** Distribution of DRG scores between DRG clusters. **(E)** mRNA levels of 14 DRGs between groups. **(F)** Distribution of DRG scores among three gene clusters. ns, not significance, **p< 0.01, ***p< 0.001, ****p< 0.0001.

First, prognosis-related DEGs were screened out by univariate Cox regression analysis. A hierarchical clustering algorithm was then used to classify the BCa samples into three gene clusters (**Figure 4A**). We observed that DRG C1 was highly correlated with gene cluster 1 while C2 was mostly related to gene cluster 3. Consistently, BCa patients in gene cluster 1 had a worse prognosis compared to those in gene cluster 3 (**Figure 4B**). Additionally, the majority of DRGs had significantly varied expression levels among the three gene clusters (**Figure 4C**).

### 3.5 Development and validation of a disulfidptosis-related prognostic model

Based on the OS-related DEGs, a disulfidptosis-related prognostic model was established for potential clinical application. The TCGA-BLCA cohort was used as the training set whereas three GEO datasets (GSE13507, GSE32548, and GSE32894) were regarded as the testing sets. Followed by LASSO and multivariate Cox regression analysis, a model with ten genes was constructed, including DCBLD2, JAM3, CSPG4, SCEL, GOLGA8A, CNTN1, APLP1, PTPRR, POU5F1, CTSE (**Figure S3**). The DRG score of each BCa sample was: DRG score =0.03458897* DCBLD2 + 0.03469218* JAM3 + 0.05195818* CSPG4 + 0.04814716* SCEL - 0.14422347* GOLGA8A + 0.02423207* CNTN1 + 0.09220036* APLP1 - 0.05143026* PTPRR + 0.02817304* POU5F1 + 0.03568603* CTSE.

Next, we aimed to investigate the correlation between DRG clusters (C1 and C2), gene clusters (1, 2, and 3), and DRG scores. Our findings indicated that DRG scores were considerably higher in DRG C1 than in C2. Moreover, the expression of DRGs was higher in BCa patients from the high-risk group (**Figures 4D, E**). We also discovered that the DRG score in gene clusters followed a rank order of 1 > 2 > 3, as illustrated in **Figure 4F**.

**Figure 5:**
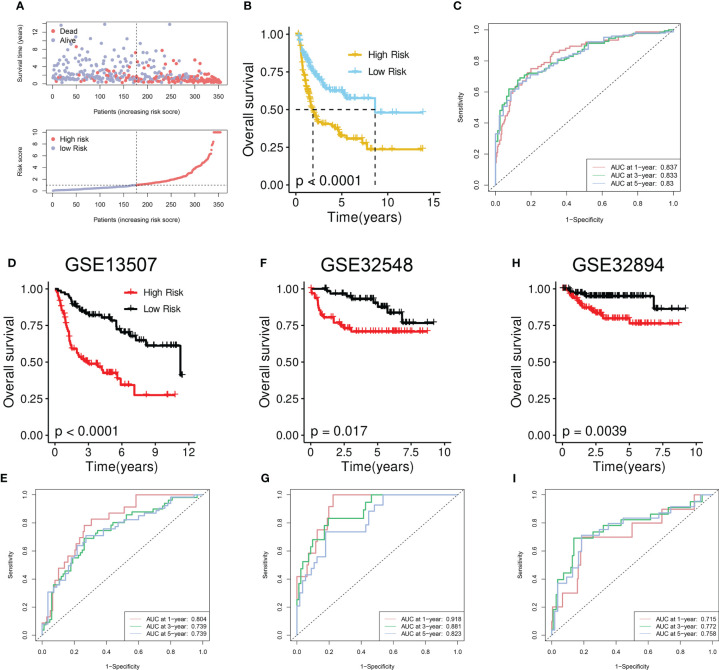
Development and validation of the disulfidptosis-related prognostic model. **(A)** Upper panel: Survival time and status between risk groups. The red dots represent dead BCa patients whereas the others indicate the alive. Bottom panel: Distribution of the DRG scores in TCGA-BLCA cohort. **(B)** Survival analysis between risk groups. **(C)** Prognostic performance of the model. **(D, F, H)** Survival analysis between risk groups in the GSE13507 **(D)**, GSE32548 **(F)**, and GSE32894 **(H)**. **(E, G, I)** Prognostic performance of the model in GSE13507 **(E)**, GSE32548 **(G)**, and GSE32894 **(I)**.

As illustrated in the risk plot, the DRG score was linked to higher mortality and decreased survival (**Figure 5A**). BCa patients of the high-risk group had poorer OS than those of the low-risk group (**Figure 5B**). Time-dependent ROC analysis showed excellent performance of the model in predicting the prognosis: AUC of 0.837, 0.833, and 0.83 at 1-, 3-, and 5-year time points, respectively (**Figure 5C**).

To further verify the performance, we tested it on three external validation cohorts. Similarly, a declined survival was observed in BCa patients of the high-risk group (**Figures 5D, F, H**). High AUC value from time-dependent ROC analysis also suggested the prognostic performance of the established model (**Figures 5E, G, I**).

### 3.6 Estimation of DRG score in immune infiltration

The biological features of BCa samples in high- and low-risk groups were assessed by GSEA (**Figures S4A, B**). The analysis revealed that cellular structure-related activities such as external encapsulating structure organization and collagen fibril organization, as well as inflammation-related GO-BP including leukocyte migration, inflammatory response, and neutrophil chemotaxis, were highly activated in the high-risk group. Conversely, metabolism-related biological processes, such as long-chain fatty acid and arachidonic acid metabolic processes, were suppressed in the high-risk group (**Figure S4A**). Furthermore, the results of GSEA on cancer hallmarks indicated that a high DRG score was linked to cell cycle (G2M checkpoint and E2F target), tumor progression (EMT, hypoxia, and angiogenesis), as well as inflammation and immunity (inflammatory response and TNFA signaling *via* NF-kappa B) (**Figure S4B**).

TIME scores including immune, stromal, and ESTIMATE scores were remarkably elevated in the high-risk group (**Figure S4C**). The correlation between immune infiltration and ten model genes was assessed (**Figure S4D**). Macrophages showed a positive correlation with DRG score, whereas CD8+ T cells, follicular helper T cells (Tfh cells), and Tregs exhibited a negative correlation with DRG score (**Figure S4E**).

Cancer stem cells possess self-renewal, multipotent, and tumor-initiating abilities, leading to tumor growth, recurrence, and resistance to current treatments. To evaluate the relationship between the DRG score and the CSC index, both were combined, revealing a weak negative correlation (**Figure S4F**).

### 3.7 Correlations between DRG score and ICGs and tumor mutational burden

Patients in the high-risk group (52%) exhibited a higher frequency of TP53 mutations than those in the low-risk group (45%) (**Figures S5A, B**). Additionally, Patients in the high-risk group had significantly higher TMB scores compared to those in the low-risk group (**Figure S5C**).

Given the reported association between ICB therapy and the expressions of ICGs, we next evaluated the association between the ICGs and DRG score. Our findings indicated a significant negative correlation between most ICGs and the ten model genes (**Figure S5D**). Specifically, the expressions of PVR, CD276, and SIRPA increased as the DRG score increased, while the expressions of TNFRSF14 and CD96 had the opposite trends (**Figure S5E**).

### 3.8 Evaluation of the model in ICB-treated cohorts

**Figure 6:**
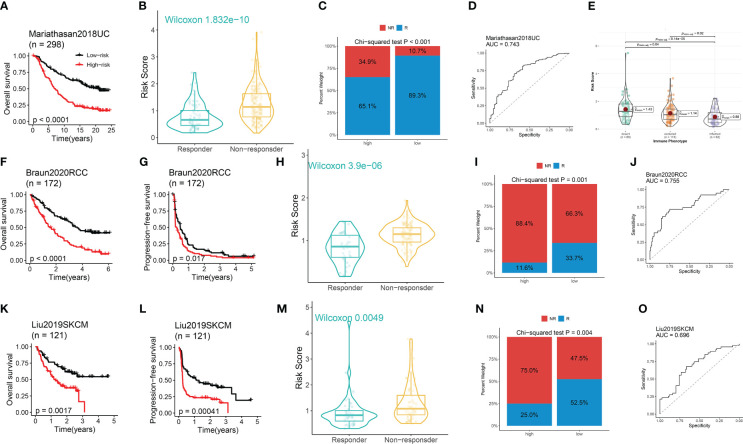
The performance of the model in predicting immunotherapeutic response in ICB-treated cohorts. **(A)** Survival analysis between risk groups. **(B)** Distribution of the DRG score between responders and non-responders. **(C)** Distribution of the responders and non-responders between risk groups. **(D)** The immunotherapeutic response prediction performance of the model. **(E)** Distribution of the DRG score among three immune phenotypes. **(A–E)** Data was analyzed in the Mariathasan cohort. **(F, K, G, I)** The Kaplan–Meier OS **(F, K)** and PFS **(G, I)** curves between risk groups. **(H, M)** Distribution of the DRG score between responders and non-responders. **(I, N)** Distribution of the responders and non-responders between high- and low-risk group. **(J, O)** The immunotherapeutic response prediction performance of the model. **(F-J)** Data was analyzed in the Braun RCC cohort. **(K-O)** Data was analyzed in the Liu SKCM cohort.

Our previous findings demonstrated a potential correlation between DRG scores and the expression of ICGs. To further investigate the predictive ability of our developed model in immunotherapy response, we analyzed three ICB-treated cohorts comprising different types of tumors. Within the Mariathasan cohort, the high-risk group was associated with a worse prognosis (**Figure 6A**). Non-responders had significantly higher risk scores compared to responders (**Figure 6B**), and the high-risk group had a lower proportion of responders and a higher proportion of non-responders than the low-risk group (**Figure 6C**). The ROC analysis revealed a high predictive performance of the model (**Figure 6D**). Additionally, the risk score gradually decreased from the desert immune phenotype to the inflamed phenotype (**Figure 6E**).

Within the Braun cohort, renal cell carcinoma (RCC) patients in the high-risk group suffered from worse OS and PFS (**Figures 6F, G**). Non-responders had significantly higher risk scores compared to responders (**Figure 6H**), and the high-risk group had a lower proportion of responders and a higher proportion of non-responders compared to the low-risk group (**Figure 6I**). The ROC analysis uncovered a high predictive performance of the model (**Figure 6J**). Similar findings were observed in the Liu cohort of patients with skin cutaneous melanoma (SKCM) (**Figures 6K–O**).

### 3.9 Correlation between DRG score and CRGs and chemotherapeutic sensitivity

Except for MGMT and AKR1C1, a negative correlation was observed between the expressions of CRGs and the ten model genes (**Figure S6A**). Additionally, EZH2, EGFR, TBX5, and SOX2 were positively correlated with the DRG score (**Figure S6B**). These findings suggested that the DRG score may be able to predict the chemotherapeutic response. To verify the findings, drug sensitivity analysis was performed using six frequently used chemotherapeutic agents in BCa patients. The results demonstrated that BCa patients in the high-risk group exhibited resistance to cisplatin, doxorubicin, gemcitabine, methotrexate, paclitaxel, and vinblastine (**Figure S6C**).

### 3.10 POU5F1 transactivates CTSE by directly binding to its promoter

**Figure 7:**
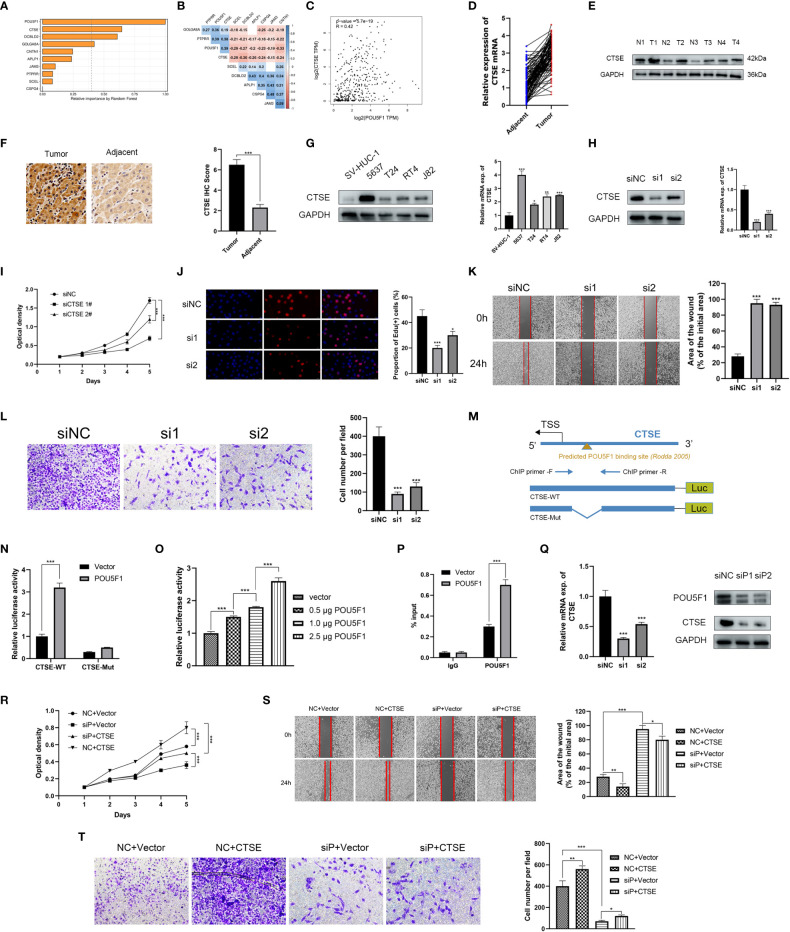
POU5F1 promoted BCa proliferation and metastasis by transactivating CTSE. **(A)** The relative importance of ten model genes. **(B)** Correlation among the ten model genes. **(C)** Spearman correlation between POU5F1 and CTSE by GEPIA. **(D, E)** Relative expression levels of CTSE in BCa tumor and adjacent normal tissues. **(F)**. Representative IHC images of CTSE in BCa tissues and adjacent normal tissues. **(G)** Expression levels of CTSE in several BCa cell lines by qRT-PCR and immunoblotting. **(H)** Immunoblotting and qRT-PCR validated the knockdown efficacy of siRNAs targeting CTSE in 5637 cell line. **(I, J)** Deficient CTSE inhibited 5637 proliferation as indicated by CCK-8 **(I)** and EdU **(J)** assays. **(K, L)** Deficient CTSE inhibited 5637 migration **(K)** and invasion **(L)**. **(M)** Diagram illustrated the predicted binding site of POU5F1 to CTSE promoter. **(N)** Luciferase activity of the POU5F1/CTSE promoter reporter was examined. **(O)** Luciferase assay with different doses of POU5F1 overexpression plasmid. **(P)** ChIP analysis indicated the enrichment of POU5F1 on the gene promoter region of CTSE. **(Q)** qRT-PCR and immunoblotting detected CTSE expression following POU5F1 depletion. **(R)** Rescuing CCK-8 assays. **(S)** Rescuing wound-healing assays. **(T)** Rescuing transwell invasion assays. **(N-T)** Assays were performed in 5637 cells. *p< 0.05, **p< 0.01, ***p< 0.001.

The random survival forests (RSF) algorithm was utilized to ascertain the relative importance of each gene in the model. As presented in **Figure 7A**, POU5F1 displayed the highest degree of significance, followed by CTSE, DCBLD2, and GOLGA8A. Our correlation analysis indicated that POU5F1 was positively correlated exclusively with CTSE (R = 0.39, **Figure 7B**). Furthermore, this result was consistent with the outcomes obtained through analysis of the GEPIA online tool (**Figure 7C**). POU5F1, also known as OCT4, is a transcriptional factor that has been linked with tumor proliferation, migration, and therapy resistance (26). Previous research has thoroughly explored the oncogenic phenotypes of POU5F1 in various human cancers, including BCa (27). Additionally, Fristrup et al. examined the protein levels of CTSE (Cathepsin E) in a large, multicenter cohort and found that CTSE levels were significantly associated with progression to stage T2-T4 BCa (28). Nonetheless, the functional roles of CTSE in BCa remain poorly understood. In light of the positive correlation between POU5F1 and CTSE, we hypothesized that POU5F1 might transactivate CTSE to promote BCa progression.

First, we investigated the expression levels of CTSE in clinical BCa tissues and BCa cell lines. In 106 pairs of BCa tissues, we observed an upregulation of both CTSE mRNA and protein levels in tumor tissues (**Figures 7D, E**), which was further supported by representative IHC images (**Figure 7F**). In particular, CTSE was found to be overexpressed in BCa cell lines, especially in 5637 (**Figure 7G**). To gain insights into the biological functions of CTSE in BCa, a series of phenotypic assays were conducted in 5637 cells. We verified the efficacy of CTSE knockdown using qRT-PCR and immunoblotting assays (**Figure 7H**). CTSE deficiency was found to inhibit 5637 cell line proliferation (**Figures 7I, J**), as well as wound-healing migration and transwell invasion assays (**Figures 7K, L**). Overall, the upregulation of CTSE drives the progression of BCa cells *in vitro*.

Next, we investigated the regulatory mechanisms between the transcription factor POU5F1 and the gene CTSE. A previous study reported the existence of a transcriptional binding site for POU5F1 (29). We then identified a potential binding site for POU5F1 at the promoter of CTSE (**Figure 7M**). To investigate the functional significance of this binding site, we conducted luciferase assays in 5637 cells. We found that co-transfection of POU5F1 significantly stimulated the luciferase activity of the CTSE-WT promoter (**Figure 7N**). Moreover, increasing the dose of POU5F1 resulted in a gradual enhancement of luciferase activity (**Figure 7O**). Next, a ChIP assay was employed to validate the enrichment of POU5F1 on the promoter of CTSE (**Figure 7M**) and found that exogenous POU5F1 promoted more POU5F1 enrichment on the CTSE promoter compared to the vector group (**Figure 7P**). Our results demonstrate that POU5F1 transactivates CTSE by directly binding to its promoter, which has regulatory effects on both mRNA and protein levels (**Figure 7Q**).

### 3.11 POU5F1 promotes BCa proliferation and metastasis by transactivating CTSE

We examined the functional association between POU5F1 and CTSE through phenotypic assays as POU5F1 was capable of regulating CTSE by physically binding to its promoter. The CCK-8 assay demonstrated that overexpression of POU5F1 enhanced the proliferation of 5637, while deficiency of CTSE inhibited it (**Figure 7R**). Similar outcomes were observed in wound-healing migration (**Figure 7S**) and transwell invasion assays (**Figure 7T**). In summary, our findings suggested that POU5F1 boosts the proliferation and metastasis of BCa cells by transactivating CTSE.

## 4 Discussion

Cell death is a crucial factor in regulating tumor proliferation (30). Previous research has established a strong association between cell death and cancer cell metabolism (31). However, the underlying mechanisms linking cell death and metabolism in BCa remain poorly understood.

Disulfidptosis, initially proposed by Xiaoguang Liu, has provided new insights into the role of disulfides and glucose metabolism dysregulation in cell death. However, the landscapes of DRGs in BCa remain unclear. In this study, we conducted a systematic investigation of the genetic and transcriptional changes of 14 DRGs in BCa. We also established a disulfidptosis-related prognostic model with 10 features which exhibited excellent performance in predicting prognosis and immunotherapeutic response.

By deciphering the genetic and transcriptional landscapes of DRGs in BCa, we found that only 27.05% of BCa samples had genetic mutations in DRGs. Surprisingly, six DRGs (ACTB, DSTN, FLNA, IQGAP1, MYL6, and TLN1) were downregulated in BCa compared to normal tissues, while CD2AP and INF2 were upregulated. Most of these downregulated DRGs (except for IQGAP1) were overexpressed in high-grade tumor tissues. Additionally, mRNA levels of ACTB, DSTN, FLNA, and TLN1 increased gradually with the tumor stage. Besides, protein levels of these genes were higher in BCa tumor tissues than in normal tissues, indicating post-translational modifications (32–35).

Based on the expression pattern of DRGs, two disulfidptosis-related molecular clusters of the TCGA-BLCA cohort were identified. BCa patients in DRG C1 were characterized by decreased survival and advanced clinicopathological features. Further decoding of the TIME unraveled that C1 was featured by the inflamed TIME in terms of upregulated expression profiles of ICGs, elevated TIME scores, and infiltrated immune cell subsets. As evidenced by previous BCa studies, tumors with distinct TIME landscapes may hold different sensitivities to chemotherapy and immunotherapy (7, 17, 36, 37). Consistently, the expression levels of CRGs and ICGs varied significantly between DRG clusters, indicating varied therapeutic responses between DRG clusters. These findings indicated the importance of disulfidptosis in driving BCa.

Further, a disulfidptosis-related prognostic model was developed based on the OS-DEGs of the DRG clusters. Deciphering the TIME of BCa unveiled that a high DRG score was linked to the inflamed phenotype which had significant effects on the cancer treatment. Furthermore, a higher RNA stemness score was detected in the high-risk group, indicating the crucial role of disulfidptosis patterns in maintaining BCa tumors.

Using the RFS method, we identified an exclusively positive correlation between two critical genes, POU5F1 and CTSE. POU5F1, also known as OCT4, encodes a transcription factor regulating stem cell pluripotency *via* the POU homeodomain. POU5F1 regulates the characteristics of tumor-initiating cells in terms of survival, self-renewal, resistance to drugs, and EMT (38). Roundhill et al. studied the role of POU5F1 in Ewing sarcoma and found that POU5F1 intensely interacted with stemness and chemoresistance genes (39). Mitchell and colleagues addressed the critical roles of POU5F1 in WDR5-induced glioblastoma progression (40). As for CTSE (Cathepsin E), its biological characteristics have been addressed in various tumors including pancreatic cancer (41), prostate cancer (42), and gastric cancer (43). In our study, we observed higher expression levels of CTSE at both mRNA and protein levels in BCa tumor tissues. Moreover, overexpression of CTSE promoted the proliferation, migration, and invasion of BCa cells. Mechanistically, we found that POU5F1 directly binds to the promoter of CTSE, leading to its transactivation and promotion of BCa progression.

## 5 Conclusion

In the study, we comprehensively investigated the DRG profiles in BCa and established a disulfidptosis-related prognostic model which exhibited excellent performance in predicting prognosis and immunotherapeutic response. BCa sample of different DRG scores was also characterized by distinct TIME landscape, response to immunotherapy and chemotherapy, and dysregulated pathways and biological processes. Furthermore, we found that POU5F1 and CTSE were critical components of the prognostic model. We also conducted further investigations to uncover the regulatory mechanisms underlying the relationship between POU5F1 and CTSE.

## Data availability statement

This data can be found here: TCGA-BLCA: https://portal.gdc.cancer.gov/GSE13507: https://www.ncbi.nlm.nih.gov/geo/query/acc.cgi?acc=GSE13507 GSE32548: https://www.ncbi.nlm.nih.gov/geo/query/acc.cgi?acc=GSE32548 GSE32894: https://www.ncbi.nlm.nih.gov/geo/query/acc.cgi?acc=GSE32894.

## Ethics statement

The studies involving human participants were reviewed and approved by Institutional Ethics Committee of Peking Union Medical College Hospital. The patients/participants provided their written informed consent to participate in this study.

## Author contributions

Conceptualization, HC and ZJ. Methodology, HC. Software, HC. Validation, HC. Formal analysis, HC. Investigation, HC. Resources, WY. YL, and LM. Data curation, WY, YL, and LM. Writing—original draft preparation, HC. Writing—review and editing, HC. Visualization, HC. Supervision, WY, YL, LM, and ZJ. Project administration, ZJ. All authors contributed to the article and approved the submitted version.

## Supplementary material

Supplementary Figure 1 Transcriptional and protein changes of DRGs. **(A)** mRNA levels of DRGs between low- and high-grade BCa tumor tissues. **(B)** mRNA levels of DRGs among stage II, III, and IV. **(C)** The protein levels of ACTB, DSTN, FLNA, and TLN1 between tumor and normal tissues by the Human Protein Atlas.

Supplementary Figure 2 DRG-based molecular clusters with distinct clinicopathological features. **(A)** The consensus score matrix when clustering number was 2. **(B)** The CDF curves of each clustering number. **(C)** The proportion of ambiguous clustering (PAC) score indicated the optimal clustering number of 2. **(D–F)** The distribution of age groups (> 65 and ≤ 65) **(D)**, gender **(E)**, and stage **(F)** between DRG-based clusters.

Supplementary Figure 3 LASSO regression.

Supplementary Figure 4 The correlation between the DRG score and TIME score. **(A, B)** The activated or suppressed GO-BP **(A)** and hallmarks **(B)** in DRG C1. **(C)** The correlations between TIME scores and DRG scores. **(D)** The correlations between the ten model genes and infiltration of 22 immune cell subsets. **(E, F)** Correlations between the DRG score and the abundances of immune cells **(E)** and the stem index **(F)**.

Supplementary Figure 5 Genetic variations and ICGs. **(A, B)** The mutation features of BCa patients in low- **(A)** and high-risk **(B)** groups. **(C)** Distribution of TMB between risk groups. **(D)** Correlations between ICGs and ten model genes. **(E)** Correlations between ICGs and the DRG score.

Supplementary Figure 6 The correlation between DRG score and CRGs. **(A)** The correlations between ten model genes and CRGs. **(B)** The correlations between CRGs and the DRG score. **(C)** Predicted IC50 between risk groups.

Supplementary Table 1 The details of included datasets.

Supplementary Table 2 The details of the 14 DRGs, DEGs between DRG-based molecular clusters, and OS-DEGs.

Supplementary Table 5 The lists of ICGs and CRGs.
