# Supplementary material for: Leveraging a disulfidptosis-based signature to improve the survival and drug sensitivity of bladder cancer patients
Source: Front Immunol. 2023 May 30;14:1198878. doi: 10.3389/fimmu.2023.1198878 (PMC10266281; doi:10.3389/fimmu.2023.1198878)
Supplement: Supplementary Figure 1 — Transcriptional and protein changes of DRGs. (A) mRNA levels of DRGs between low- and high-grade BCa tumor tissues. (B) mRNA levels of DRGs among stage II, III, and IV. (C) The protein levels of ACTB, DSTN, FLNA, and TLN1 between tumor and normal tissues by the Human Protein Atlas. [file DataSheet_1.pdf]

Supplementary Material

Leveraging a disulfidptosis-based signature to improve the survival and drug sensitivity of bladder cancer patients

Hualin Chen, Wenjie Yang, Yingjie Li, Lin Ma, Zhigang Ji*

*** Correspondence:** Zhigang Ji   jizhigang@pumch.cn

1 Supplementary Figures and Tables

1.1 Supplementary Figures

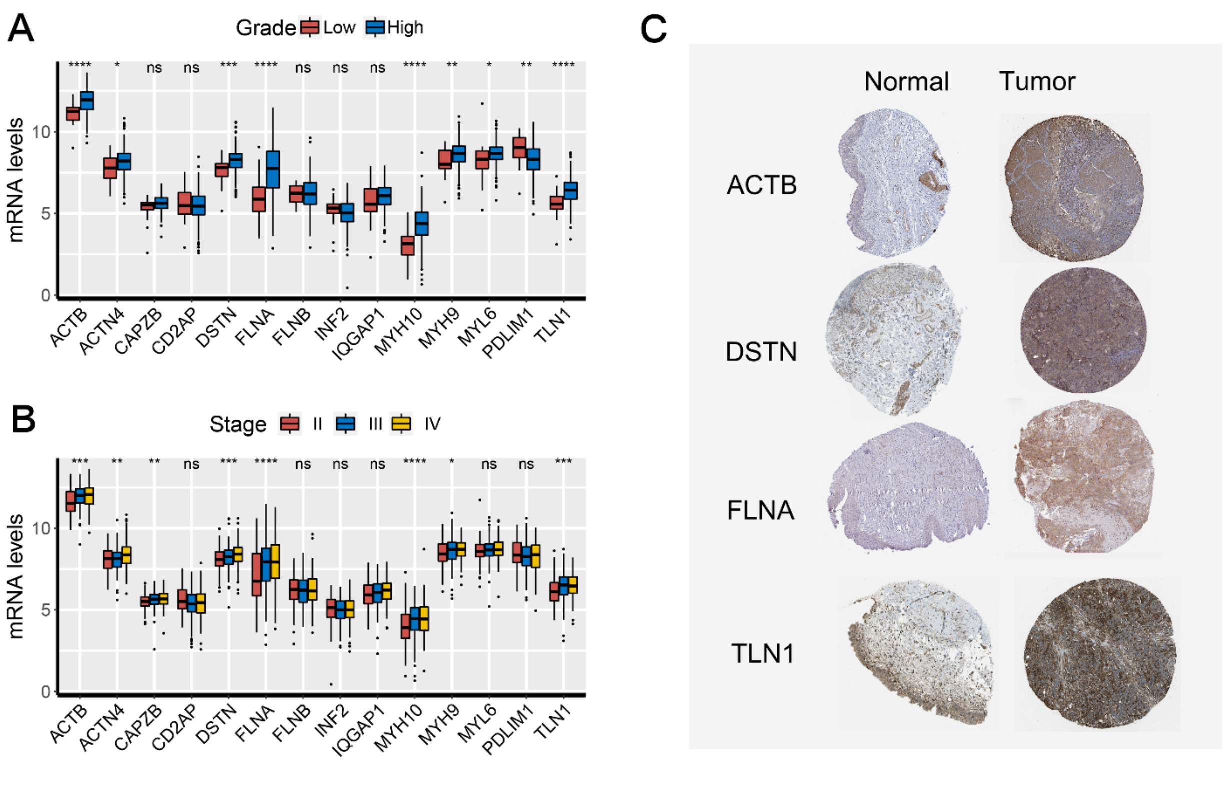

Figure S1 Transcriptional and protein changes of DRGs. **A** mRNA levels of DRGs between low- and high-grade BCa tumor tissues. **B** mRNA levels of DRGs among stage II, III, and IV. **C** The protein

levels of ACTB, DSTN, FLNA, and TLN1 between tumor and normal tissues by the Human Protein Atlas.

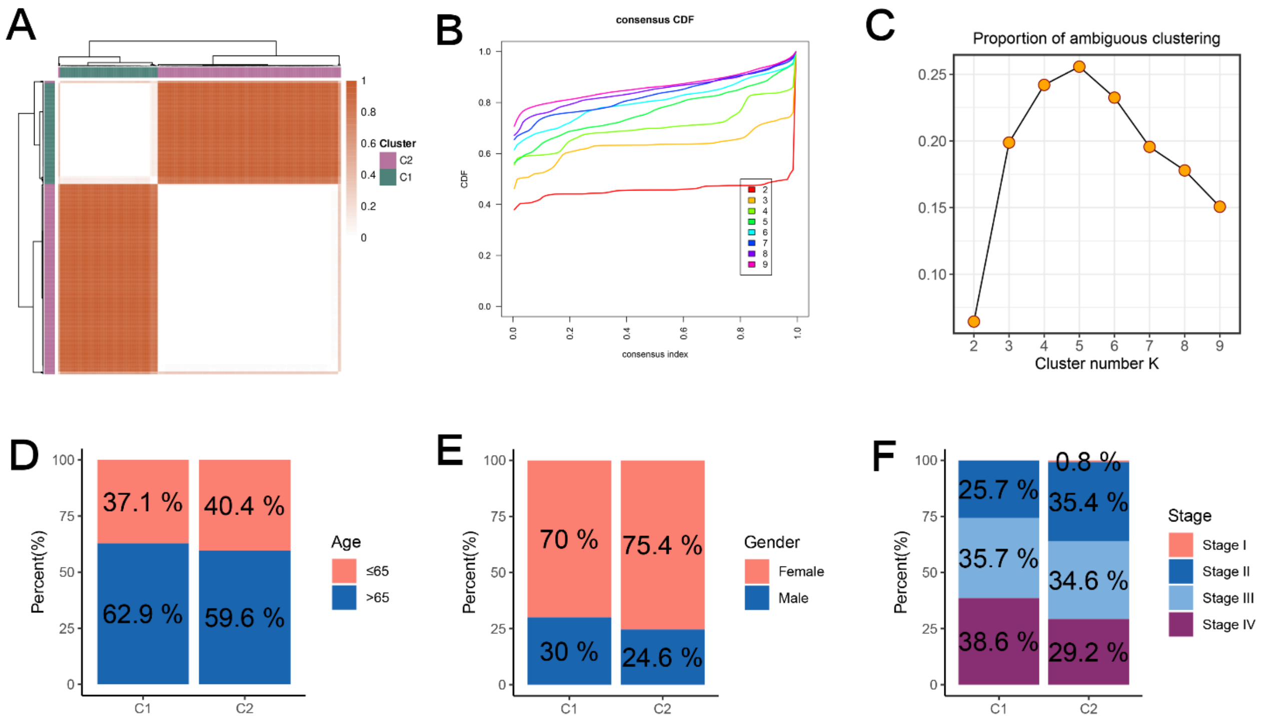

Figure S2 DRG-based molecular clusters with distinct clinicopathological features. **A** The consensus score matrix when clustering number was 2. **B** The CDF curves of each clustering number. **C** The proportion of ambiguous clustering (PAC) score indicated the optimal clustering number of 2. **D-F** The distribution of age groups (> 65 and ≤ 65) (**D**), gender (**E**), and stage (**F**) between DRG-based clusters.

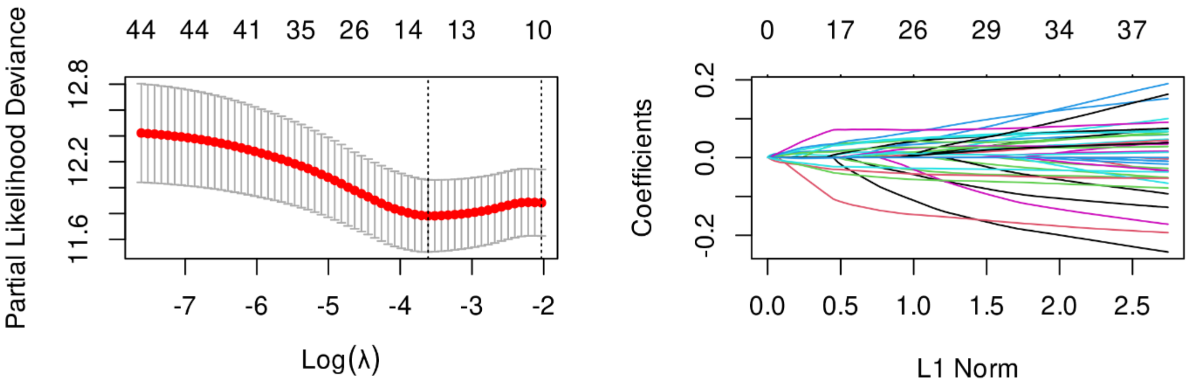

Figure S3 LASSO regression.

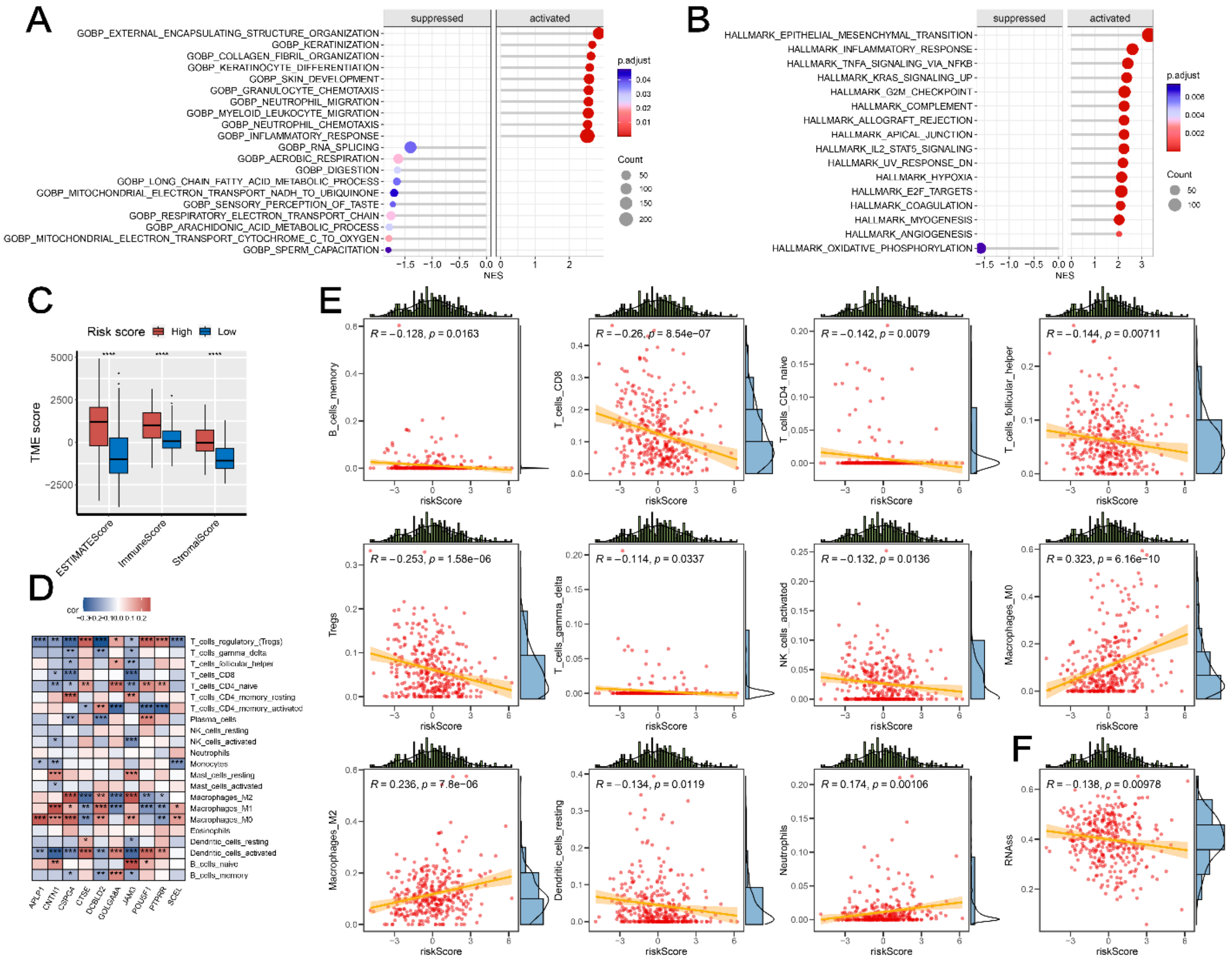

Figure S4 The correlation between the DRG score and TIME score. **A, B** The activated or suppressed GO-BP (**A**) and hallmarks (**B**) in DRG C1. **C.** The correlations between TIME scores and DRG scores. **D** The correlations between the ten model genes and infiltration of 22 immune cell subsets. **E, F** Correlations between the DRG score and the abundances of immune cells (**E**) and the stem index (**F**).

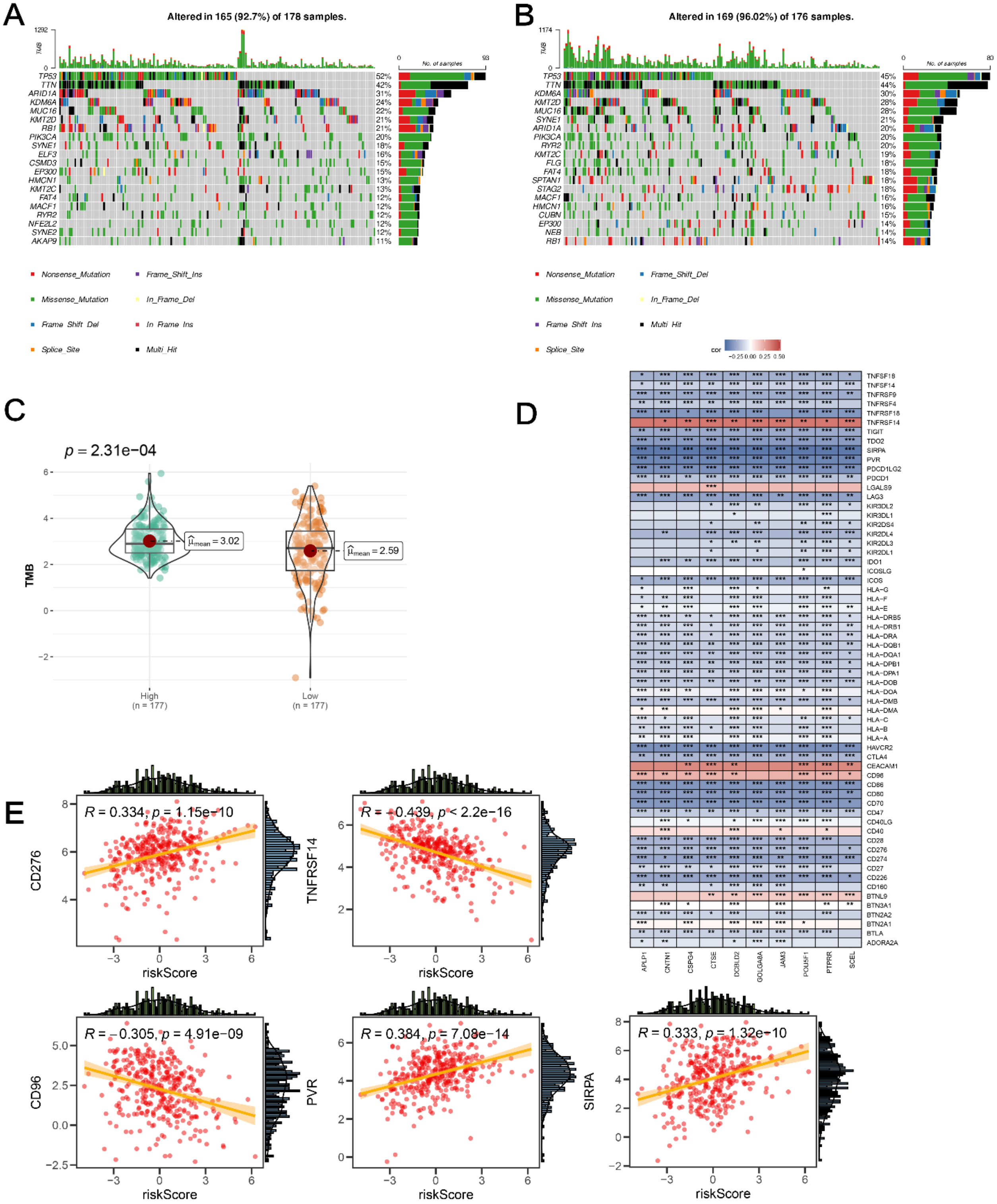

A
Altered in 165 (92.7%) of 178 samples.
B
Altered in 169 (96.02%) of 176 samples.
Nonsense_Mutation
Missense_Mutation
Frame_Shift_Del
Splice_Site
Frame_Shift_Ins
In_Frame_Del
In_Frame_Ins
Multi_Hit
C
p = 2.31e−04
TMB
High (n = 177)
Low (n = 177)
D
cor
E
R = 0.334, p = 1.15e−10
R = −0.439, p < 2.2e−16
R = −0.305, p = 4.91e−09
R = 0.384, p = 7.08e−14
R = 0.333, p = 1.32e−10
CD276
TNFRSF14
CD96
PVR
SIRPA
riskScore

Figure S5 Genetic variations and ICGs. (**A, B**) The mutation features of BCa patients in low- (**A**) and high-risk (**B**) groups. (**C**) Distribution of TMB between risk groups. (**D**) Correlations between ICGs and ten model genes. (**E**) Correlations between ICGs and the DRG score.

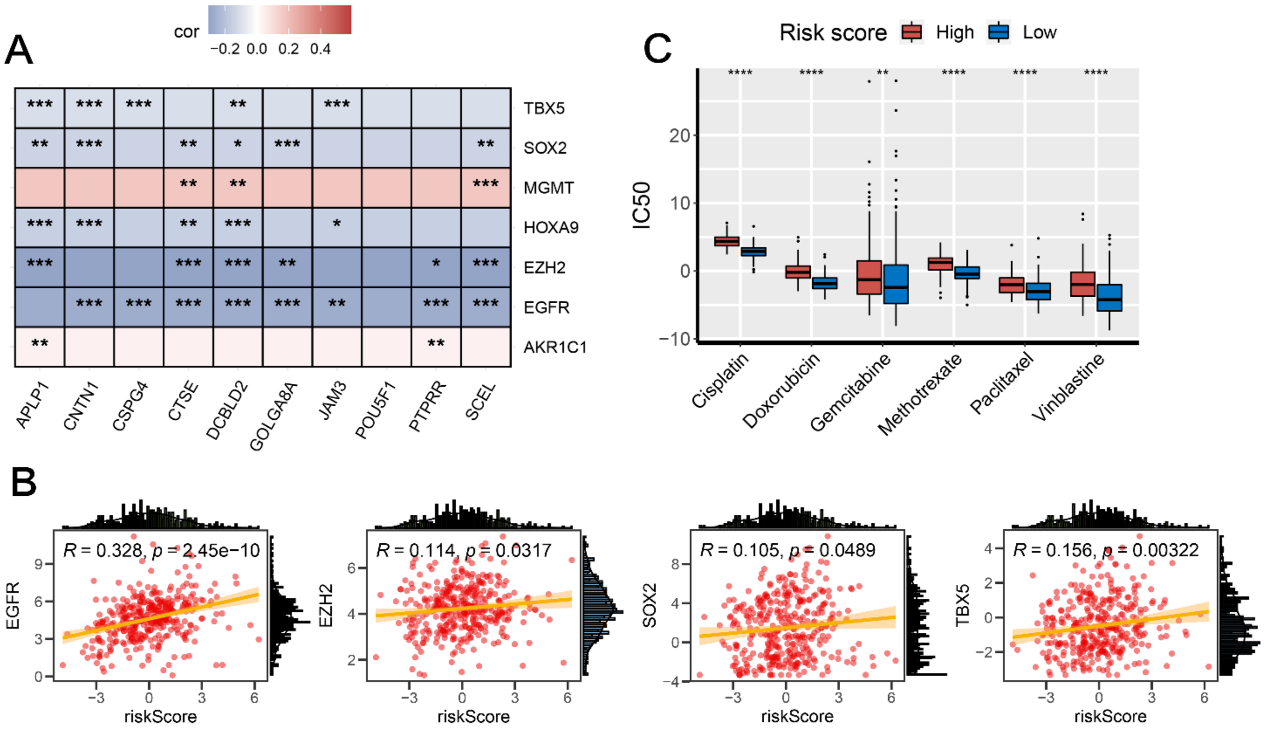

Figure S6 The correlation between DRG score and CRGs. **A** The correlations between ten model genes and CRGs. **B** The correlations between CRGs and the DRG score. **C** Predicted IC50 between risk groups.

1.2 Supplementary Tables

Table S1 The details of included datasets.

Table S2 The details of the 14 DRGs, DEGs between DRG-based molecular clusters, and OS-DEGs

Table S3 Primers used in this study.

Table S4 Antibodies used in this study.

Table S5 The lists of ICGs and CRGs.
