# Supplementary material for: Leveraging a disulfidptosis-based signature to improve the survival and drug sensitivity of bladder cancer patients
Source: Front Immunol. 2023 May 30;14:1198878. doi: 10.3389/fimmu.2023.1198878 (PMC10266281; doi:10.3389/fimmu.2023.1198878)
Supplement: Supplementary Table 5 — The lists of ICGs and CRGs. [file Table_3.docx]

**Table S3. Primers used in this study.**

| **Gene** | **Forward sequence (5' – 3')** | **Reverse sequence (5' – 3')** |
| --- | --- | --- |
| qRT-PCR primers | | |
| CTSE | CATACAGCCAGCCAGGTCAA | GCTCCAATGATCCCGGACAA |
| ChIP-qPCR primers | | |
| CTSE | TTGTCCGGGATCATTGGAGC | CACAGCCAAGGAGGGGTATC |
