# Supplementary material for: Leveraging a disulfidptosis-based signature to improve the survival and drug sensitivity of bladder cancer patients
Source: Front Immunol. 2023 May 30;14:1198878. doi: 10.3389/fimmu.2023.1198878 (PMC10266281; doi:10.3389/fimmu.2023.1198878)
Supplement: Supplementary file 5 [file Table_4.docx]

**Table S4. Antibodies used in this study.**

| **Antibodies** | **Catalog number** | **Company** | **Dilution ratio** |
| --- | --- | --- | --- |
| CTSE | ab151880 | Abcam | 1:1000 |
| POU5F1 | 11263-1-AP | Proteintech | 1:2500 |
| GAPDH | 60004-1-Ig | Proteintech | 1:50000 |
